# Association between early‐life farming exposure and Alzheimer's disease neuropathological burden

**DOI:** 10.1002/alz.71904

**Published:** 2026-10-09

**Authors:** W. Ryan Powell, Eleanna M. Melcher, Rachel C. Otte, Sarah Lim, Lauren Etter, Aly Pfaff, Amanda DeWitt, Adrienne R. S. Lee, Fred B. Ketchum, M. Shahriar Salamat, Christopher A. Bradfield, Stephen A. Goutman, Megan L. Zuelsdorff, Kelly M. Bakulski, Robert A. Rissman

**Affiliations:** ^1^ Center for Health Disparities Research University of Wisconsin School of Medicine and Public Health Madison Wisconsin USA; ^2^ Department of Medicine Geriatrics Division University of Wisconsin School of Medicine and Public Health Madison Wisconsin USA; ^3^ Department of Population Health Sciences University of Wisconsin School of Medicine and Public Health Madison Wisconsin USA; ^4^ Department of Medical Physics University of Wisconsin School of Medicine and Public Health Madison Wisconsin USA; ^5^ Department of Neurology University of Wisconsin–Madison School of Medicine and Public Health Madison Wisconsin USA; ^6^ Department of Neurological Surgery University of Wisconsin School of Medicine and Public Health Madison Wisconsin USA; ^7^ Department of Pathology and Laboratory Medicine University of Wisconsin School of Medicine and Public Health Madison Wisconsin USA; ^8^ Molecular & Environmental Toxicology Center University of Wisconsin Madison Wisconsin USA; ^9^ Biotechnology Center University of Wisconsin Madison Wisconsin USA; ^10^ Department of Neurology University of Michigan Ann Arbor Michigan USA; ^11^ University of Wisconsin School of Nursing Madison Wisconsin USA; ^12^ Wisconsin Alzheimer's Disease Research Center University of Wisconsin School of Medicine and Public Health Madison Wisconsin USA; ^13^ University of Michigan School of Public Health Ann Arbor Michigan USA; ^14^ Michigan Alzheimer's Disease Research Center University of Michigan Medical School Ann Arbor Michigan USA; ^15^ Department of Physiology and Neuroscience Alzheimer's Therapeutic Research Institute Keck School of Medicine of the University of Southern California San Diego California USA

**Keywords:** Alzheimer's disease risk, amyloid positivity, farming, neurofibrillary pathology, neuropathology, occupational exposome, occupational risk factors, social exposome

## Abstract

**BACKGROUND:**

Farming populations may face a disproportionate share of adverse exposures influencing Alzheimer's disease (AD), yet farming data are rarely connected to cohorts with characterized AD neuropathology. Whether early‐life exposure is related to AD neuropathology is limited.

**METHOD:**

Brain donors from two Alzheimer's Disease Research Centers (*N* = 1035; between 1986–2018) were included. US Census records were used to identify those with early‐life farming exposure (birth to 17 years old) and examined alongside AD neuropathology assessments.

**RESULT:**

Early‐life farming exposure (≈ 17% of donors) was associated with 60% higher odds of AD neuropathological burden (odds ratio = 1.60, 95% confidence interval: 1.09–2.35) adjusting for age, sex, and year of death.

**CONCLUSION:**

Findings suggest exposure to farming early in life is linked to increased levels of AD neuropathology. Deeper occupational and residential phenotyping is needed to identify the potential multiple, overlapping risk pathways, which may include cumulative and sensitive life stage exposure to chemical toxicants, hearing loss, and psychosocial hazards.

## Highlights

- Farmers may be a high‐risk group for developing Alzheimer's disease (AD).
- Farming data are rarely connected to brain donation AD studies.
- This study included people donating their brains to science over a 30‐year period.
- Our team identified who had early‐life farming histories according to Census data.
- Experts looked for signs of AD in the brains of these individuals.
- We found farming early in life is linked to increased signs of AD in the brain.
- This is the first study to report this finding when examining AD neuropathology.
- Research needs to figure out the specific ways that early‐life farming impacts AD.

## 1 INTRODUCTION

The occupational exposome measures all the working conditions and contexts that a person experiences during their life and may include fundamental drivers of Alzheimer's disease (AD) development, yet such exposomal data are rarely included in AD neuropathology (ADNP) research studies, limiting our understanding of modifiable AD risk factors and mechanistic pathways of disease development. Certain experiences, including exposure to adverse environmental and social factors, may create high‐risk occupational groups facing a disproportionate burden of AD risk.

In particular, farming populations may face a disproportionate share of risk factors (known and potentially yet to be discovered) including agriculture chemical exposure (e.g., pesticides^1^, ^2^, ^3^, ^4^, ^5^ and heavy metals^1^, ^6^), psychosocial hazards^7^, ^8^, ^9^, ^10^ (e.g., stress and its downstream factors), and physical stressors (e.g., hearing loss).^11^, ^12^, ^13^, ^14^, ^15^, ^16^ In addition, whether adverse exposure during potential sensitive periods, including early life, is associated with increased levels of AD neuropathological burden measured at autopsy is not well understood. The developing brain during early and adolescent life is vulnerable to environmental and social influences.^17^, ^18^, ^19^, ^20^ Yet there is insufficient evidence to support a direct link between farming and AD development, with new data on this topic limited to non‐neuropathological studies.^21^, ^22^ While such studies have advanced our understanding of the link between work and AD and related dementias (ADRD), few existing AD studies link gold‐standard brain tissue neuropathology to occupational exposome metrics. Insight into early‐life influences could lead to new exposomal discoveries and the development of targeted prevention and intervention strategies that minimize risks and provide efficient allocation of resources to those standing to benefit the most at certain times. The objective of this study is to leverage occupational records systematically collected during donors’ early lives to characterize farming exposure and study its association with AD neuropathologic change at autopsy.

**RESEARCH IN CONTEXT**

1. **Systematic review**: The occupational exposome measures all work‐related exposures throughout one's lifetime. Farming populations may face a disproportionate share of known and unknown Alzheimer's disease (AD) risk factors, yet such data are rarely connected to gold standard AD neuropathology assessments at autopsy. Whether adverse farming exposure during potential sensitive periods, including early life, is associated with increased levels of AD neuropathological burden measured at autopsy is not well understood.
2. **Interpretation**: This is the first study to suggest a link between early‐life farming exposure and elevated levels of AD neuropathological burden measured at autopsy.
3. **Future directions**: There are multiple overlapping pathways by which early‐life farming may influence AD risk. Future research should involve deeper social and environmental phenotyping and exposomal analysis to identify the specific farming exposure pathways that underlie this association, which will require novel data collection methods for brain banks.

## 2 METHODS

### 2.1 Sample

The study sample includes individuals who donated to one of two Alzheimer's Disease Research Center (ADRC) brain bank programs between 1986 and 2018 with neuritic plaque density and neurofibrillary tangles staging characterized by a neuropathologist (*n* = 1153). Donors were excluded if they did not have at least one decennial publicly available US Federal Census record from early life (between birth and 17 years of age)—70 (59.3% of those excluded) lived outside the United States in their early life (i.e., no information collected by Census), 30 (25.4%) were born after the 1950 Census recording (i.e., records were not systematically available), 18 (15.3%) were unable to be located in Census records despite living in the United States during early life and Census records should have been systematically available. Institutional review boards at the University of Wisconsin–Madison and the University of California San Diego determined that the project did not constitute human subjects research. Data exchanges between collaborating institutions were conducted under Health Insurance Portability and Accountability Act–compliant data use agreements to ensure the protection of all donor information.

### 2.2 Early‐life farming exposure

To measure early farming exposure, we leveraged the Population and Housing Census, a component within the US decennial census, which is systemically recorded by trained census experts (i.e., enumerators) every 10 years and broadly available for individuals living in the United States during early life (available publicly up through 1950). Early farming exposure was derived from systematic extractions of information using records from early life (birth through 17 years of age; see Appendix S1 in supporting information for specific Census fields).

Donors are assigned as having farming exposure if any of their early life census records indicate the following:

1. Working on a farm: a farming‐related occupation was reported in the “Occupation, Industry, and Class of Workers” section by anyone in the household (Figures S1–S2 in supporting information).
2. Living on a farm: an affirmative response to the census item indicating whether donor's home was on a farm (Figures S3–S8 in supporting information).

Data abstraction and validation were guided by unstructured medical record abstraction standards and the Genealogical Proof Standard. ^23^, ^24^, ^25^ Early‐life farming exposure was abstracted by two trained researchers using public data tracing methods established previously:^26^, ^27^ (1) independent abstraction by the first archivist, (2) verification of the identity and content of each record, (3) independent search by the second archivist, and (4) joint resolution to resolve any disagreements. Researchers systematically searched digital and paper archival collections available both in ‐person and online (e.g., Ancestry.com) to locate records. Data were extracted between February 2024 and October 2025. This is a novel approach to exposure science and ADRD risk analysis to include the occupational exposome factors via public data extraction, overcoming the challenge of new data collection when prospective data collection (e.g., via survey methods) is no longer possible.

### 2.3 Neuropathology data

Neuropathologic data were obtained either from the National Alzheimer's Coordinating Center (NACC) Neuropathology Data Form, or through systematic abstraction of information from autopsy reports for donors whose autopsies occurred prior to the implementation of standardized NACC neuropathology protocols. Two trained abstractors independently extracted all variables using a detailed abstraction manual developed with expert neuropathologist input. Interrater reliability was high (Braak stage, Cohen kappa = 0.99, and amyloid C score = 0.98), and disagreements were resolved by team consensus. Neuritic plaque density was categorized using Consortium to Establish a Registry for Alzheimer's Disease criteria (none, sparse, moderate, frequent), and neurofibrillary tangle burden was staged using Braak criteria (B0: no tangles; B1: Braak I/II, transentorhinal stages; B2: Braak III/IV, limbic stages; B3: Braak V/VI, isocortical stages). These measures correspond to the C and B components of the National Institute on Aging–Alzheimer's Association ABC scoring framework for AD neuropathologic change.^28^ Because A scores (Thal phase for amyloid plaques) were not available for the majority of the sample (systematic collection began in 2014), the outcome of ADNP was defined using an established definition^29^ as having both moderate to frequent neuritic plaques and Braak stage III to VI.

### 2.4 Statistical analysis

Descriptive statistics for demographic, early‐life farming, and neuropathologic features were calculated and are reported in Table 1. Using generalized logistic regression, we modeled the association between autopsy‐confirmed ADNP and early‐life farming (key explanatory variable) adjusting for variables related to AD risk and regularly available across the three‐decade donation time period: age at death (linear and quadratic terms), sex, and year of death ≥ 2002 (i.e., year that the National Alzheimer's Coordinating Center introduced standardized measurement of neuropathology data collection and reporting activities using the Minimum Data Set at ADRCs) with site‐clustered standard errors.

**TABLE 1:** Brain bank donor sample characteristics.

Characteristic	Overall sample (*n* = 1035)	ADNP^a^ (*n* = 766)	No ADNP (*n* = 269)
Early life farming exposure – no. (%)	173 (16.7)	141 (18.4)	32 (11.9)
Mean age – year ± SD	81.5±8.7	81.4±8.2	81.6±10.1
Sex – no. (%)			
Female	494 (47.7)	385 (50.3)	109 (40.5)
Male	541 (52.3)	381 (49.7)	160 (59.5)
Year of death – no (%)^b^			
1986 to 2001	290 (28.0)	214 (27.9)	76 (28.3)
2002 to 2018	745 (72.0)	552 (72.1)	193 (71.7)

Abbreviations: ADNP, Alzheimer's disease neuropathology; SD, standard deviation.

^a^ ADNP defined as both moderate to frequent neuritic plaques and Braak stage III–VI) per established definition.

^b^ 2002 is the year that the National Alzheimer's Coordinating Center began implementing standardized neuropathology data collection and reporting (the Neuropathology Data Set) across Alzheimer's Disease Research Centers.

Those excluded from the analysis were significantly younger (*M *= 75.1 years of age, 95% confidence interval [CI: 72.6 to 77.7] vs. *M *= 81.5, 95% CI [80.9 to 82.0], *P *< 0.001) but otherwise not different on sex (47.5% vs. 52.3%, *P *= 0.322) or year of death ≥ 2002 (78.8% vs. 72.0%, *P *= 0.115). Modeling was conducted using Stata/SE 19.5 (StataCorp LLC). The analysis was conducted from June 1, 2025 to March 18, 2026. Strengthening the Reporting of Observational Studies in Epidemiology (STROBE) reporting guidelines were followed.

## 3 RESULTS

The sample consisted of 1035 donors spanning 31 years of brain donation (48% female, 81.5 mean age, median birth 1924 [interquartile range (IQR) = 1918–1931] and death year 2009 [IQR = 2000–2013]; Table 1). Approximately 17% (*n* = 173) of the sample reported early‐life farming exposure and 74% (*n* = 766) had ADNP. In modeled analysis, early‐life farm exposure was associated with a 60% increased adjusted odds of ADNP burden (Table 2; odds ratio [OR] = 1.60, 95% CI: 1.09–2.35, *P *= 0.017) relative to those without exposure. Additional analysis suggests that *both* living and working on a farm early in life confers 98% higher odds compared to *only* living (16.7% of donors) or only working on a farm (< 1% of donors) in early life (Table S1 in supporting information; OR = 1.98; 95% CI: 1.94–2.02, *P *< 0.001).

**TABLE 2:** Adjusted odds of Alzheimer's disease neuropathology associated with early life farming exposure.

ADNP^a^		
Characteristic	Crude OR	Adjusted OR
Early Life Farming Exposure		
No	1 (Reference)	
Yes	1.67 (1.05 to 2.66), *P* = 0.031	1.60 (1.09 to 2.35), *P* = 0.017
Age (Linear)		1.67 (1.55 to 1.81), *P <* 0.001
Age (Quadratic)		1.00 (1.00 to 1.00), *P* < 0.001
Sex		
Female		1 (Reference)
Male		0.62 (0.49 to 0.78), *P* < 0.001
Year of death ≥ 2002^b^		1.07 (0.64 to 1.81), *P* = 0.792

*Notes*: Cell values denote OR and site clustered 95% CIs produced from generalized logistic regression models.

Adjusted model includes all variables listed in table

(age, sex, and year of death).

Abbreviations: ADNP, Alzheimer's disease neuropathology; CI, confidence interval; OR, odds ratio.

^a^ ADNP defined as both moderate to frequent neuritic plaques and Braak stage III to VI per established definition.

^b^ Categorized before and after 2002 because that is the year that the National Alzheimer's Coordinating Center began implementing standardized neuropathology data collection and reporting (the Neuropathology Data Set) across Alzheimer's Disease Research Centers.

## 4 DISCUSSION

Our findings underscore the potential adverse impact of early‐life occupational exposome factors on AD developed later in life. The multiple, overlapping pathways by which early‐life farming exposure may influence AD risk are uncertain, but may include agriculture chemicals (e.g., pesticides,^1^, ^2^, ^3^, ^4^, ^5^ heavy metal exposure^1^, ^6^), hearing loss,^11^, ^12^, ^13^, ^14^, ^15^, ^16^ and psychosocial strain.^7^, ^8^, ^9^ Results cannot determine which specific aspects of farming are driving the observed association with AD neuropathological risk. There are likely several biologically plausible pathways involved, many of which remain untested and underexplored in ADNP research. For example, pesticides are a known neurotoxicant causing mitochondrial impairment, oxidative stress resulting in cell damage, and neuronal cell death.^30^, ^31^, ^32^ The relationship between pesticides and Parkinson's disease is well established,^33^, ^34^ while AD is less understood.^35^, ^36^, ^37^, ^38^, ^39^, ^40^ Furthermore, high levels of heavy metal compounds (e.g., lead, arsenic) were commonly used in the early to mid‐twentieth century agriculture ecosystems and can contaminate soil for decades if not remediated, are elevated in farming populations.^1^ Interactions are also likely but currently under‐researched. For example, heavy metal exposure during childhood may contribute to hearing loss,^12^, ^14^ and farmers are more likely to experience hearing loss than other occupational groups. Heavy metal exposure may have a role in both central auditory processing dysfunction and higher levels of AD‐related neuropathology burden through shared biological mechanisms.^41^ Whether hearing loss—a modifiable mid‐life risk factor for AD and cognitive decline—partially mediates the association between early‐life heavy metal exposure and later life AD risk, or reflects shared underlying AD‐related biological processes (e.g., tau pathology) is unknown.^13^, ^15^, ^16^, ^41^

Future brain bank research should involve deeper occupational phenotyping and related exposomal analysis in national generalizable samples to identify the specific farming exposure pathways that underlie this association. For example, greater and more precise environmental and social characterization of agricultural activities and locations across the full life course (including prenatal exposure^19^) may reveal where risks are most elevated. Recent epidemiological evidence examining diagnosis and medication use data to identify AD cases among the French agricultural workforce^21^ suggests crop, viticulture, fruit, and arboriculture farmers face increased risk. However, these associations have not been examined in relation to gold standard–verified ADNP assessments or other biological endpoints. For brain bank research, this information will need to be categorized using other novel data collection methods because systematic individual‐level farm data collected by Agriculture Census enumerators were aggregated for statistical reporting (e.g., state, county level) and subsequently destroyed.^42^

Additional ways to link the occupational exposome to biological endpoints are also needed. For brain bank research, next steps should also include more precise measures of environmental exposures and directly measuring tissue‐level chemical concentrations at autopsy for proxy of lifetime accumulation. Active participation in a cohort study during donor lives involving additional biomonitoring devices and biospecimens measurement of exposure is also vital for life‐course analysis. Additional exposomal dimensions should be considered for robust exposomal analysis. Early‐life farming may reflect the complex interplay of factors across the exposome, including life‐course socioeconomic conditions, education, rural residence, cohort/period effects, and lifelong occupational trajectories, all of which may influence later life AD risk. These factors should be examined alongside early‐life farming characteristics to gain deeper insight. Future research should also assess cumulative exposure as early‐life exposure may extend into adulthood for additional insight into the influence of dosage and timing beyond adolescence.

## CONFLICT OF INTEREST STATEMENT

The authors declare no conflicts of interest. Author disclosures are available in the Supporting Information.

## CONSENT STATEMENT

All donors consented to brain donation for the purposes of advancing Alzheimer's disease and related dementias research. This study was reviewed by institutional review boards at participating ADRC sites and was determined to be not human subjects research; as such, informed consent was not required.

## Supporting information




Supporting Information

Supporting Information
