# Supplementary material for: Association between early‐life farming exposure and Alzheimer's disease neuropathological burden
Source: Alzheimers Dement. 2026 Oct 9;22(10):e71904. doi: 10.1002/alz.71904 (PMC13650515; doi:10.1002/alz.71904)
Supplement: Supplementary file 2 [file ALZ-22-e71904-s002.docx]

**Appendix**

**Supporting Information**

**Title:** Association Between Early Life Farming Exposure and Alzheimer’s Disease Neuropathological Burden

**Authors:** W. Ryan Powell; Eleanna M. Melcher; Rachel C. Otte; Sarah Lim; Lauren Etter; Aly Pfaff; Amanda DeWitt; Adrienne Lee; Fred B. Ketchum; M. Shahriar Salamat; Christopher A. Bradfield; Stephen A. Goutman; Megan L. Zuelsdorff; Kelly M. Bakulski; Robert A. Rissman

Table of Contents

Appendix 1. Early life farming exposure flag methods and data sources	2

Supplementary Table 1. Adjusted odds of Alzheimer’s neuropathology associated with early life farming exposure (both living and working vs. only working or only living)	7

**Appendix 1: Early Life Farming Exposure Flag Methods and Data Sources**

Study individuals were expected to have one to two census assessments during early life depending on year of birth relative to census date. To ensure completeness of early-life census capture, we calculated the *expected* number of census records for each donor based on birth year relative to federal enumeration dates. Donors with fewer records than expected were flagged for targeted additional searching using variant name spellings, alternative family members, and additional geographic leads. After completing all searches, early life census records were identified for 1,035 individuals (n=753 with two expected records; n=233 with a single expected record; n=49 with one of two expected records).

***Figure A1.*** *Census Population Schedules Record indicating farming occupation and industry (example is from 1930 but is similarly arranged for all other Census versions 1900-1940)*

***Figure A2.*** *1950 Census Population Schedules Record indicating a non-farming occupation and industry*

***Figure A3.*** *1900 Census Population Schedules Record columns indicating residence on farm.* *Reported living on a farm if column 27 = ‘F’/’Fm’/‘Farm’ & presence of farm schedule digit in column 28; reported not living on a farm if column 27 = ‘H’ for house & absence of farm schedule digit in column 28*

***Figure A4.*** *1910 Census Population Schedules Record columns indicating residence on farm. Reported living on a farm if column 28 = ‘F’/’Fm’/‘Farm’ & presence of farm schedule digit in column 29; reported not living on a farm if column 28 = ‘H’ for house & absence of farm schedule digit in column 29*

***Figure A5.*** *1920 Census Population Schedules Record columns indicating residence on farm. Reported living on a farm if column 2 = ‘F’/’Fm’/‘Farm’ & presence of farm schedule digit in column 29; reported not living on a farm if column 2 = a house number/‘X’ for a house that has no number & absence of farm schedule digit in column 29*

***Figure A6.*** *1930 Census Population Schedules Record column indicating residence on farm. Reported living on a farm if column 10 = ‘Y’/‘Yes’; reported not living on a farm if column 10 = ‘N’/’No’*

***Figure A7a and A7b.*** *1940 Census Population Schedules Record columns indicating residence on farm. Reported living on a farm if column 6 = ‘Y’/‘Yes’ OR column 20 = ‘Y’/‘Yes’ for 1935 residence (1935 residence only used if donor between birth-17 at this timepoint); reported not living on a farm if column 6 = ‘N’/‘No’ AND column 20 = ‘N’/‘No’*

***Figure A8****. 1950s Census Population Schedules Record column indicating residence on farm. Reported living on a farm if column 4 = ‘Y’/‘Yes’; reported not living on a farm if column 4 = ‘N’/‘No’*

Supplementary Table 1. Adjusted odds of Alzheimer’s neuropathology associated with early life farming exposure (both living and working vs. only working or only living)

| **Alzheimer's** **Disease** **Neuropathology** **(ADNP)^a^** | | |
| --- | --- | --- |
| **Characteristic** | **Crude** **OR** | **Adjusted** **OR** |
| Early Life Farming Exposure |  |  |
| Living Only *or* Working Only | 1 (Reference) |  |
| Living *and* Working | 2.05 (1.59 to 2.63), *p*<0.001 | 1.98 (1.94 to 2.02), *p*<0.001 |
| Age (Linear) |  | 1.84 (1.65 to 2.05), *p<*0.001 |
| Age (Quadratic) |  | 1.00 (1.00 to 1.00), *p*<0.001 |
| Sex |  |  |
| Female |  | 1 (Reference) |
| Male |  | 0.56 (0.42 to 0.74), *p*<0.001 |
| Year of death ≥ 2002^b^ |  | 0.90 (0.73 to 1.10), *p*=0.306 |

*Notes*: Cell values denote OR and site clustered 95% CIs produced from generalized logistic regression models.

Adjusted model includes all variables listed in table

(age, sex, and year of death).

Abbreviations: ADNP, Alzheimer’s disease neuropathology; CI, confidence intervals; OR, odds ratio.

^a^ ADNP defined as both moderate to frequent neuritic plaques and Braak stage III–VI) per established definition

^b^ Categorized before and after 2002 because that is the year that the National Alzheimer’s Coordinating Center began implementing standardized neuropathology data collection and reporting (the Neuropathology Data Set) across ADRCs
